# Objective scoring of application forms in obstetrics and gynaecology residency selection: A retrospective cohort study on the optimal number of committee members

**DOI:** 10.1371/journal.pone.0336478

**Published:** 2025-11-19

**Authors:** Wim J. R. Rietdijk, Janneke K. Oostrom, Petra C. A. M. Bakker

**Affiliations:** 1 Department of Institutional Affairs, Vrije Universiteit Amsterdam, Amsterdam, The Netherlands; 2 Department of Hospital Pharmacy, Erasmus University Medical Center, Rotterdam, The Netherlands; 3 Department of Social Psychology, Tilburg School of Social and Behavioral Sciences, Tilburg University, Tilburg, The Netherlands; 4 Department of Obstetrics and Gynaecology, Amsterdam UMC, Amsterdam, The Netherlands; Washington University in St. Louis School of Medicine, UNITED STATES OF AMERICA

## Abstract

**Introduction:**

The selection of residents for medical specialty programmes is a critical yet resource-intensive process. Although structured evaluation tools, such as standardized application forms, enhance objectivity and reliability, they often require all committee members to assess every candidate, resulting in inefficiencies. This study aimed to determine the optimal number of assessors needed to reliably score application forms of doctors to become resident in obstetrics & gynaecology without compromising selection outcomes.

**Methods:**

This retrospective cohort study analysed data from three residency selection cycles (of the years 2022–2024) in the Northwest region of the Netherlands. Application forms were scored anonymously each year by 15–18 committee members, referred to as assessors, using a structured scoring system. Scores were analysed to identify the point at which adding more assessors no longer significantly impacted candidate rankings. Statistical measures included paired t-tests, correlations, and Cronbach’s alpha, and intraclass correlation coefficients to assess internal consistency and reliability.

**Results:**

The analysis showed that six assessors are sufficient to reliably assess candidates. Correlations between average scores from six assessors and the grand average consistently exceeded 0.9 across all cohorts, and Cronbach’s alpha stabilized above 0.85. Significant differences in rankings were observed when increasing assessors from two to six but diminished beyond six. Bland-Altman plots confirmed agreement between scores from six assessors and the overall committee evaluation.

**Conclusion:**

A structured evaluation process (i.e., using standardized application forms) requiring six assessors per candidate ensures reliable and consistent outcomes while reducing workload. Implementing this approach can enhance efficiency without compromising fairness or objectivity in selection for obstetrics & gynaecology residents. Future research should investigate the applicability of this model to other medical residency programmes internationally, and its impact on long-term performance.

## Introduction

Resident selection is a critical step in ensuring the quality of postgraduate training and, ultimately, patient care. In many specialties, including Obstetrics & Gynaecology (OBGYN), selection procedures are organized regionally and typically involve a two-stage process, sometimes preceded by a national or central application round: an initial screening followed by interviews or assessment rounds [1]. Traditionally, the first selection is often based on applicants’ curriculum vitae (CV, or resume), motivational letters, and letters of recommendation. However, there is growing evidence that unstructured assessment of CVs, motivational letters, and letters of recommendation is prone to bias and may favour applicants with stronger social networks or institutional capital, rather than merit alone [1–6].

To promote fairness, transparency, diversity, and consistency in selection, structured application forms are increasingly being used as an alternative to traditional CV-based selection in medical education [1,2,7–12]. These forms guide assessors and applicants in a more systematic and standardized evaluation of relevant competencies and experiences [7], thereby reducing bias and increasing reliability [13]. However, a key challenge in applying structured forms is managing inter-assessor variability; differences in how individual assessors interpret and score the same application. While structured tools can improve consistency and reliability [7,14], to our knowledge there are only few studies on qualitatively assessing the inter-assessor variability within the evaluation of medical students in clerkships [15], and selection of medical residents in particular [16].

At our OBGYN department, applicants are reviewed by all members of the selection committee. The annual process of selecting new residents is labor-intensive, posing challenges to sustainability in the context of a shrinking healthcare workforce and increasing clinical workload [17]. Since 2022, the selection procedure has been standardized, but the resource-intensive nature of this procedure still required evaluation. The procedure has been standardized by means of using application forms and standardized evaluation form that help guide the evaluation of the assessors (see S1 Material). This study aims to evaluate the stability of first-round applicant rankings based on structured application forms in a regional OBGYN residency selection process. Specifically, we seek to determine the minimum number of assessors required to achieve consistent and reliable outcomes. The underlying rationale is to identify the point at which additional assessors no longer contribute meaningful changes to candidate rankings—commonly referred to as the ‘saturation point’. Establishing this threshold is essential for optimizing the trade-off between fairness and resource use, particularly in the context of increasing workload and workforce shortages in healthcare. The results will inform the efficient design of transparent and equitable selection procedures in OBGYN and potentially other medical specialties.

## Methods

### Study context

The selection process for residents to become gynaecologist in the Northwest region of the Netherlands, conducted from 2022 to 2024, was evaluated in this retrospective cohort study. This region consists of eight hospitals, including one academic hospital. The number of residency positions available each year was nine in 2022 and eight in both 2023 and 2024. The number of applicants was 29 in 2022, 14 in 2023, and 21 in 2024, with 15 candidates invited for an interview each year. The selection committee typically included 15–18 members from various hospitals within the region, including gynaecologists and a few residents in OBGYN. This study was approved by the medical ethics committee of Amsterdam UMC (approval number: 2023.0732). The data was extracted from the original forms and pseudonymized by the secretary of the department. The pseudonymized data were transferred to the authors for final statistical analysis on May 20^th^, 2024. The data and analysis files are available from the corresponding author on reasonable request.

### Selection process

Interested candidates submitted their CV and a motivation letter in response to the vacancy. The programme director and residency programme administrator reviewed these documents to ensure candidates met the eligibility criteria: possession of a medical degree, at least one year of clinical experience in obstetrics and gynaecology, and support from the department of OBGYN at the last hospital where the candidate worked. Candidates who met these criteria received a link to a digital application form, which collected general information, educational background, and clinical experience. The application form also inquired about the candidate’s experience in various domains, including societal impact, education and training, technical innovation, and research.

The digital application forms were anonymized and reviewed by the selection committee members, who scored the forms on a scale from 0 (no experience) to 2 (extensive experience) for each domain. Although a guideline (S1 Material) was provided to assist committee members in scoring the forms, they had some discretion in assigning scores. For example, in the research domain, a score of 0 was given for no publications, 1 for several publications, and 2 for completing a PhD. The highest-scoring candidates were then invited for structured interviews. Here, a committee member is referred to as an “assessor”.

### Study outcome

This study focuses on the number of assessors and their rating of each candidate needed to reach the point at which adding more assessors no longer yields additional information. Therefore, the primary outcome measure was the stability of candidate selection, defined as the point at which adding additional assessors to the committee does not result in a significant change in the overall score an applicant received.

### Statistical analysis

The data are analysed in several steps. We recorded the scores assigned by each assessor for each candidate. Each year, a total of 15–18 assessors evaluated the application forms of each candidate. Using these scores, we calculated an average score. Random pairs of assessor scores were included in the averages (i.e., 2, 4, 6, 8, 10, and the grand average across all assessors), and paired t-tests were used to determine statistically significant differences. We also analysed correlations between the grand assessor score and the averages from 2, 4, 6, 8, and 10 randomly chosen assessors. Additionally, Cronbach’s alpha was calculated to assess internal consistency, which is identical to a two-way random effects intraclass correlation coefficient (ICC) [18]. The point at which adding more assessors did not yield additional information, as indicated by the stabilization of averages and the plateauing of correlation and Cronbach’s alpha values. A correlation of 0.90 was used as the cutoff, and Cronbach’s alpha of 0.85 indicated high internal consistency. At this saturation point, the average scores showed agreement with the grand average assessor score. The analysis was adjusted based on methods from Rietdijk et al., Olvet and Hajcak, and Pontifex et al. [19–21], thresholds were reported in the studies mentioned before except for the ICC, which would be considered sufficient above 0.75 [18]. Data for the three cohorts (2022, 2023, and 2024) were analysed separately.

## Results

### Baseline characteristics of the committee members

Baseline characteristics of the committee members, such as years of experience, role, gender, and whether they worked in an academic or non-academic hospital are presented in Table 1. The majority of members were female and employed in a non-academic teaching hospital. Notably, only in the first year, in 2022, an educational specialist was included as part of the committee.

**Table 1:** Baseline characteristics of the assessors.

	2022 n = 18 (100%)	2023 n = 17 (100%)	2024 n = 15 (100%)
Resident	3 (17)	3 (18)	3 (20)
Gynaecologists	14 (78)	14 (82)	12 (80)
Educational specialist	1 (6)	0 (0)	0 (0)
Male	3 (17)	3 (17)	3 (20)
Non-academic hospital	8 (44)	8 (47)	8 (53)

### Cohort 2022

**Fig 1:**
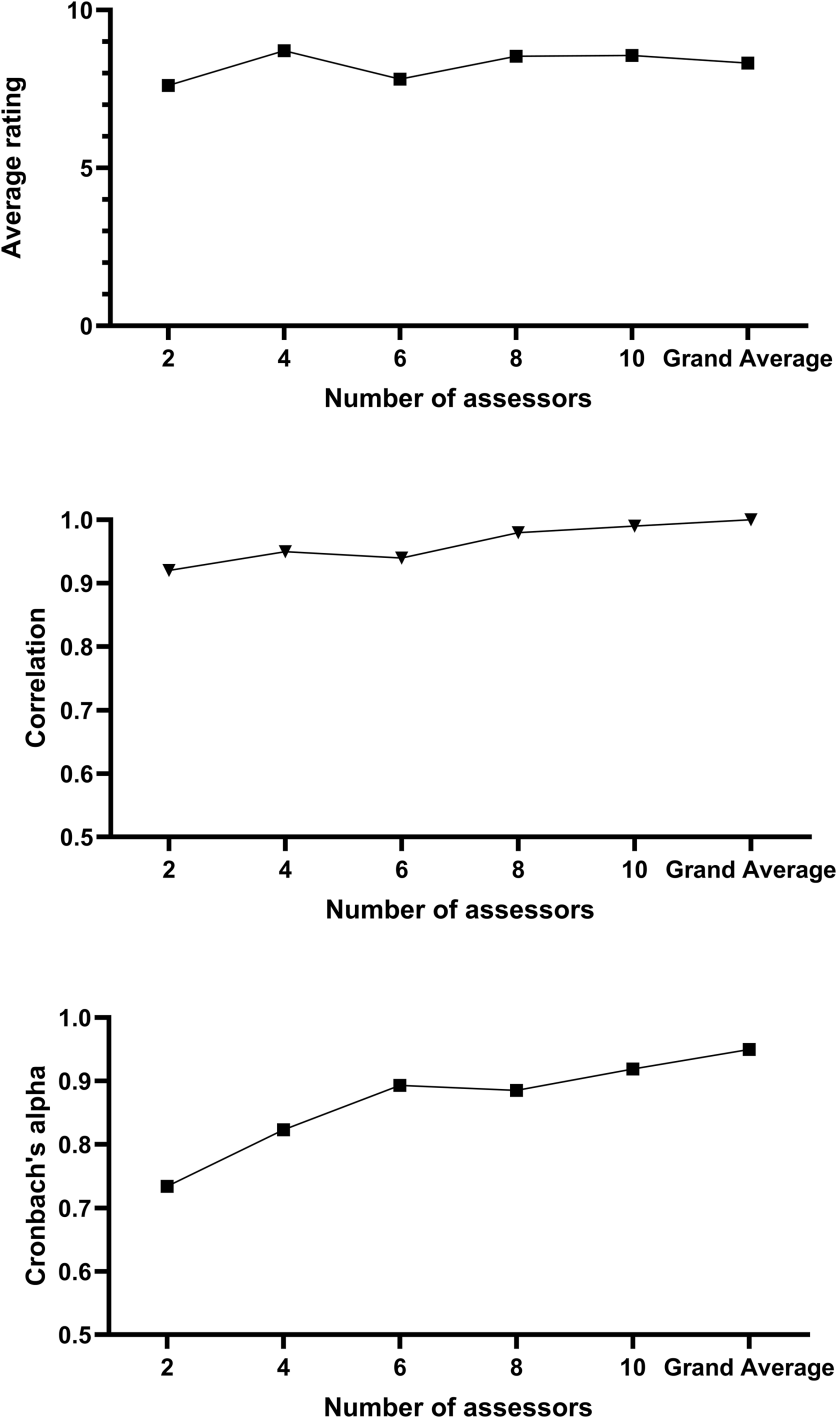
Presenting the analysis for the 2022 Cohort. In the upper panel, we show the average rating for each candidate for an increasing number of assessors (i.e., average score of 2, 4, 6, 8, and all – randomly selected – assessors). In the middle panel, we show the correlation between the average score of the increasing number of assessors and the grand average (i.e., all assessors). In the bottom panel, we present the Cronbach’s Alpha for the increasing number of assessors. The results are for the cohort 2022.

**Fig 2:**
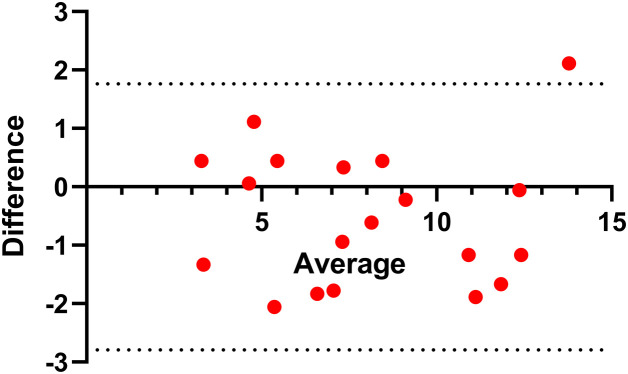
The Bland-Altman plot of agreement. The Bland-Altman shows the agreement between the average score of the evaluations by six assessors and the average score of all assessors for cohort 2023.

Fig 1 presents the results for the selection process in 2022. The upper panel shows the average scores for 2, 4, 6, 8, and 10 assessors compared to the grand average. The middle panel shows the correlation between the evaluations by random assessors (2, 4, 6, 8, and 10) and the grand average. The lower panel presents the Cronbach’s alpha for the evaluations of each pair of assessors. Significant differences in average ratings were found when comparing 2 vs. 4 assessors (p = 0.049), 4 vs. 6 assessors (p = 0.013), and 6 vs. 8 assessors (p = 0.012), while differences were not significant when comparing 8 vs. 10 assessors (p = 0.958) and 10 vs. the grand average (p = 0.073). Significant positive correlations above 0.90 (p < 0.05) were found between the average scores for 2, 4, 6, 8, and 10 assessors and the grand average. Cronbach’s alpha increased with the number of assessors, exceeding 0.85 from 6 assessors onward, the ICCs supported this cohort 2022 result (S1 Table). Fig 2 shows the Bland-Altman plot of agreement (bias = −0.52, 95% limits of agreement −2.79 to 1.76) between the evaluation by 6 assessors and the grand average, indicating agreement between the two.

### Cohort 2023

S1 Fig presents the results for the selection process in 2023. Significant differences in average ratings were found when comparing 2 vs. 4 assessors (p = 0.010) and 10 vs. the grand average (p = 0.009), while differences were not significant when comparing 4 vs. 6 assessors (p = 0.068), 6 vs. 8 assessors (p = 0.142), and 8 vs. 10 assessors (p = 0.827). Significant positive correlations above 0.880 (p < 0.05) were found between the average scores for 2, 4, 6, 8, and 10 assessors and the grand average. Cronbach’s alpha increased with the number of assessors, exceeding 0.85 from 4 assessors onward, the ICCs supported this cohort 2023 result (S1 Table). S2 Fig shows the Bland-Altman plot of agreement (bias = −0.49, 95% limits of agreement −1.57 to 0.60) between the evaluation by 6 assessors and the grand average, indicating agreement between the two.

### Cohort 2024

S3 Fig presents the results for the selection process in 2024. Significant differences in average ratings were found when comparing 2 vs. 4 assessors (p < 0.001), 4 vs. 6 assessors (p = 0.016), and 6 vs. 8 assessors (p = 0.006), while differences were not significant when comparing 8 vs. 10 assessors (p = 0.568) and 10 vs. the grand average (p = 0.427). Significant positive correlations above 0.927 (p < 0.05) were found between the average scores for 2, 4, 6, 8, and 10 assessors and the grand average. Cronbach’s alpha increased with the number of assessors, exceeding 0.85 from 4 assessors onward, the ICCs supported this cohort 2024 result (S1 Table). S4 Fig shows the Bland-Altman plot of agreement (bias = 0.23, 95% limits of agreement −0.53 to 0.99) between the evaluation by 6 assessors and the grand average, indicating agreement between the two.

## Discussion

### Principal findings

This study aimed to determine the optimal number of assessors required to reliably score application forms for medical residency selection in OBGYN, ensuring that adding more assessors would not significantly alter selection outcomes. Our findings demonstrate that six assessors are sufficient to provide stable and reliable scores, as increasing the number beyond six did not result in meaningful changes in evaluation of job application candidates. In other words, each applicant’s form has to be evaluated by six (randomly assigned) assessors, rather than all assessors to make a well-informed decision to invite an applicant for the interview round. Thereby making the selection process less labour-intensive.

### Relevance of the findings and comparison with existing literature

The study’s results provide a clear threshold for committee size in residency selection, demonstrating that six randomly chosen assessors out of a larger pool of assessors are sufficient for reliable evaluations. The correlation between their average scores and the overall committee (grand) average consistently exceeded 0.9, while internal consistency (Cronbach’s alpha) stabilized above 0.85. These metrics show that a small group of assessors can produce reliable scores, which is crucial for high-stakes decisions in medical education. Previous research has emphasized the importance of structured and objective selection tools to reduce bias and improve the reliability and validity of the selection process, and our findings align with this evidence [6,8]. Structured evaluation forms, like those used in this study, enhance objectivity and fairness by reducing subjectivity and increasing inter-assessor reliability. The high consistency observed in this study (Cronbach’s alpha > 0.85 with six assessors) further supports the effectiveness of structured methods for achieving reliable and fair outcomes in residency selection [21]. These findings offer practical guidance for reducing workload while maintaining high-quality standards in selection processes.

### Strengths and limitations

A major strength of this study is its systematic approach to evaluating assessor performance across three distinct cohorts. By analysing correlations, Cronbach’s alphas, and Bland-Altman plots, we provide robust evidence that six assessors suffice to produce reliable ratings.

However, the study is limited by its single-centre design, with data derived from one residency programme in the Netherlands. This may restrict the generalizability of the findings to other specialties or regions. However, more specialty programmes had similar set up as compared to ours. This may suggest that with additional analysis of these data one may produce similar results as compared to the present study. In turn, this may also provide evidence for a more efficient and objective selection processes of residents. Furthermore, while the study focuses on optimizing efficiency, it does not evaluate whether the six-assessor model impacts the diversity of selected candidates or the validity for predicting (long-term) performance in the residency programme. In a meta-analysis, it was found that more objective selection strategies are the most useful in association with longer term performance [22]. Yet the impact of our standardized approach on longer term performance is missing [23]. Furthermore, the inclusion of six assessors does not say anything about the – perhaps – homogeneous composition of the selection committees. When the selection committee may be more diverse, other number of the committee members may occur from the statistical analysis.

### Practical implications

Implementing a six-assessor, randomly selected model has immediate practical benefits for medical residency programmes. It reduces the workload for committee members, simplifies the logistical challenges of convening large committees, and maintains the reliability of the selection process. Assessor consistency is crucial for ensuring a fair selection process—demonstrating that candidates’ chances are not dependent on idiosyncratic biases of assessors. However, if assessors have similar backgrounds, they may still exhibit the same systematic biases. Such biases include, among other, the fact that selection committees tend to select candidates that are more similar to them. For example, male committee members are more likely to select men for jobs in general, and leadership roles in particular [24]. Hence, improving diversity may require additional systemic interventions. For example, selection committee members ought to be trained in how to address unconscious biases. Furthermore, the diversity of the selection committee itself needs to be prioritized, as diverse selection committees are more effective in fairly assessing candidates from varied backgrounds [5,25].

Systematic interventions must also address the recruitment of diverse candidate pools and on retaining diverse candidates throughout all career stages. Efforts such as mentorship programmes [5,26], pipeline initiatives [25,27], financial support [28], and inclusive admission policies [1] are essential to encourage underrepresented groups to pursue medical specialties. These strategies should complement the optimized selection process to ensure equitable opportunities and create a more representative physician workforce.

### Future research

Future studies should assess whether the six-assessor model enhances equitable candidate selection and increase the diversity of selected candidates. Additionally, research should examine the long-term performance of candidates chosen through this model and its applicability to other selection processes for residency programmes in different countries. Furthermore, comparative studies could evaluate the effectiveness of this model against alternative selection tools, such as structured application forms and interviews or AI-assisted evaluations, particularly in fostering diversity.

## Conclusion

A structured evaluation process (i.e., using standardized application forms) requiring six assessors per candidate ensures reliable and consistent outcomes while reducing workload. Implementing this approach can enhance efficiency without compromising fairness or objectivity in selection for obstetrics & gynaecology residents. Future research should investigate the applicability of this model to other medical residency programmes internationally, and its impact on long-term performance.

## Supporting information

S1 Fig Results of cohort 2023. (DOCX)

S2 Fig Bland-Altman cohort 2023. (DOCX)

S3 Fig Results of cohort 2024. (DOCX)

S4 Fig Bland-Altman 2024. (DOCX)

S1 Material Guide for assessors to rate job applicants, freely translated from the Dutch language. (DOCX)

S1 Table Intraclass correlations across all cohorts. (DOCX)
