# Supplementary material for: Objective scoring of application forms in obstetrics and gynaecology residency selection: A retrospective cohort study on the optimal number of committee members
Source: PLoS One. 2025 Nov 19;20(11):e0336478. doi: 10.1371/journal.pone.0336478 (PMC12629435; doi:10.1371/journal.pone.0336478)

Supplementary Fig 3 Results of Cohort 2024

In the upper panel, we show the average rating for each candidate for increasing number of assessors (i.e., average score of 2, 4, 6, 8, and all – randomly selected - assessors). In the middle panel, we show the correlation between the average score of the increasing number of assessors and the all assessors (i.e., grand average). In the bottom panel, we present the Cronbach’s Alpha for the increasing number of assessors. The results are for the cohort 2024.


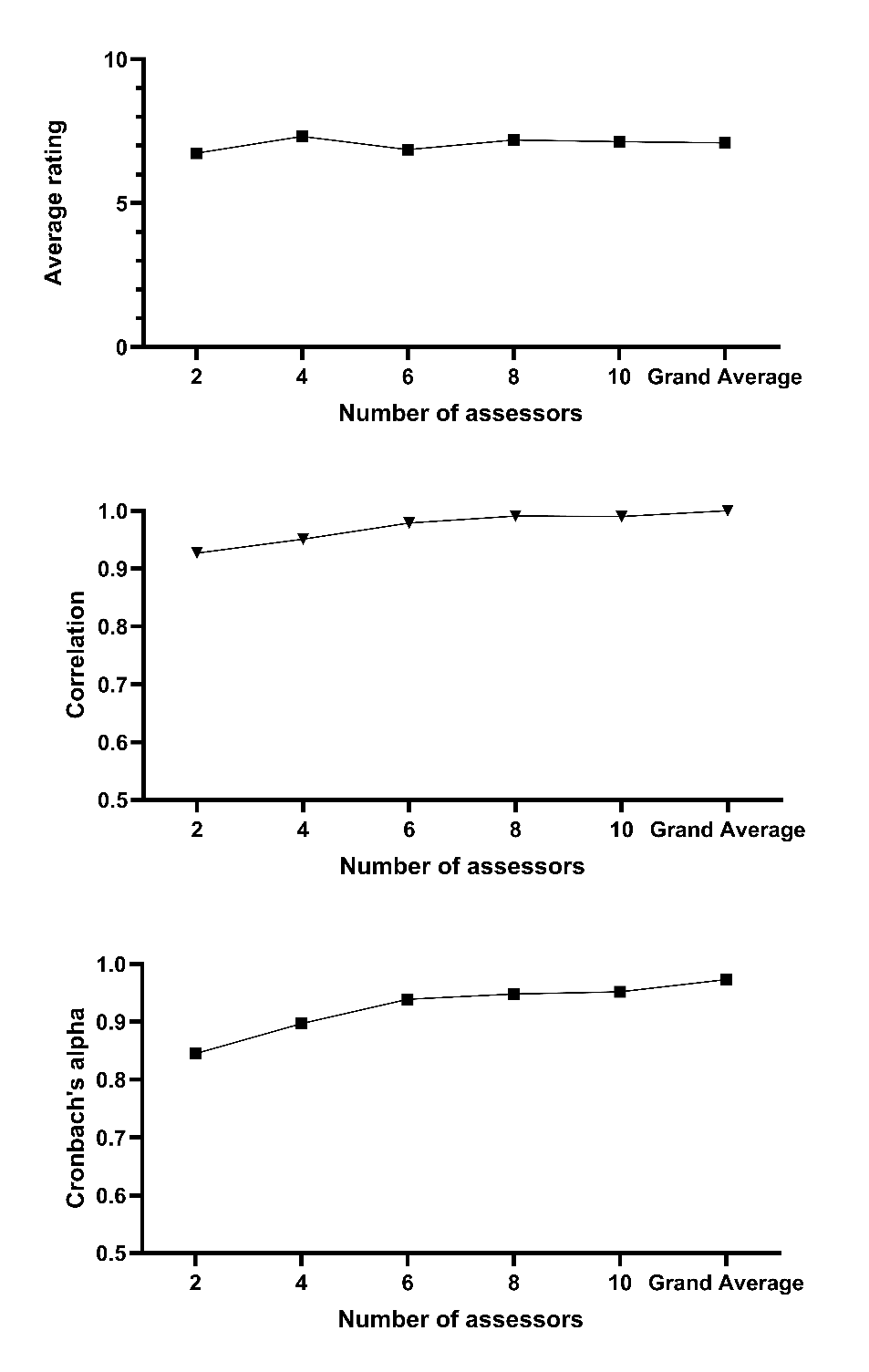

Supplement: S3 Fig — (DOCX) [file pone.0336478.s003.docx]
