# Supplementary material for: Objective scoring of application forms in obstetrics and gynaecology residency selection: A retrospective cohort study on the optimal number of committee members
Source: PLoS One. 2025 Nov 19;20(11):e0336478. doi: 10.1371/journal.pone.0336478 (PMC12629435; doi:10.1371/journal.pone.0336478)

Supplementary Fig 4 Bland-Altman cohort 2024

The Bland-Altman shows the agreement between average score of the evaluations by six assessors and the average score of all assessors for cohort 2024.


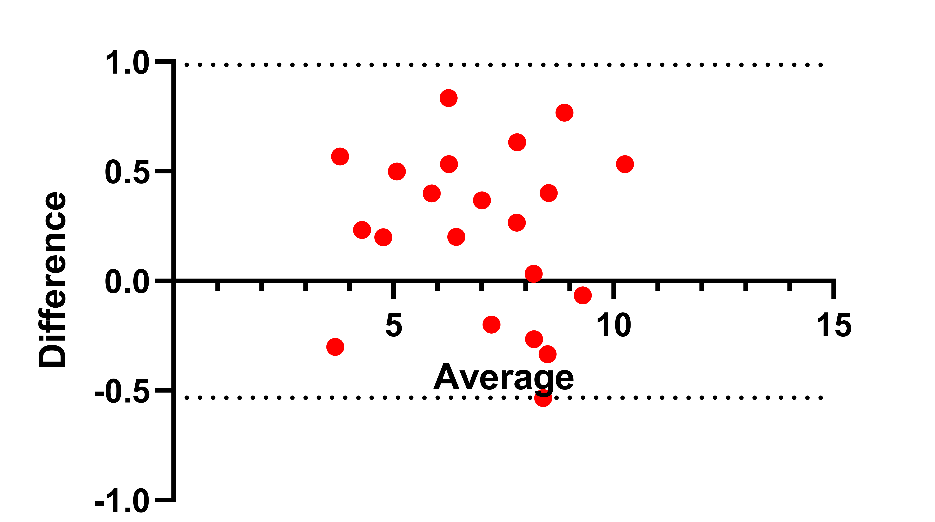

Supplement: S4 Fig — (DOCX) [file pone.0336478.s004.docx]
