# Supplementary material for: Objective scoring of application forms in obstetrics and gynaecology residency selection: A retrospective cohort study on the optimal number of committee members
Source: PLoS One. 2025 Nov 19;20(11):e0336478. doi: 10.1371/journal.pone.0336478 (PMC12629435; doi:10.1371/journal.pone.0336478)
Supplement: S1 Material — (DOCX) [file pone.0336478.s005.docx]

Supplementary Material 1 Guide for assessors to rate job applicants, freely translated from the Dutch language

**Have the candidate completed a PhD degree? *(Required)***

 Yes

 No

**How do you value the other scientific experience of the candidate *(Required)***

 The candidate has no relevant scientific experience.

 The candidate has some relevant scientific experience.

 The candidate has much relevant scientific experience.

 The candidate follows as PhD trajectory.

**How do you value the additional education programmes the candidate has completed? *(Required)***

 There is no additional education programme completed.

 There is an additional education programme completed, that is of some added value.

 There is an additional education programme completed, that is very much added value.

**How do you value the candidate’s organizational skills? *(Required)***

 The candidate has no relevant organizational skills

 The candidate has some relevant organizational skills

 The candidate has significant organizational skills.

**How do you value the teaching experience of the candidate? *(Required)***

 The candidate has no significant teaching skills.

 The candidate has some experience teaching experience.

 The candidate has significant teaching experience.

**How do you value the candidate’s experience with respect to innovation and technology? *(Required)***

 The candidate has no relevant experience with respect to innovation and technology.

 The candidate has some relevant experience with respect to innovation and technology.

 The candidate has significant relevant experience with respect to innovation and technology.

**How do you value the societal experience of the candidate *(Required)***

 The candidate has no societal experience.

 The candidate has some societal experience.

 The candidate has significant societal experience.

**To what extent does the candidate contribute to the diversity of the resident group and to the future profession / specielty *(Required)***

 The candidate does not contribute to the diversity of the resident group or future profession / specialty.

 The candidate does contribute a little to the diversity of the resident group or future profession / specialty.

 The candidate does contribute to the diversity of the resident group or future profession / specialty.

**What is your overall impression of the candidate? *Required)***

 The candidate is not suitable as a candidate for the residency program for the obstetrics gyneacology.

 The candidate is somewhat suitable as a candidate for the residency program for the obstetrics gyneacology.

 The candidate is very much suitable as a candidate for the residency program for the obstetrics gyneacology.
