# Supplementary material for: Objective scoring of application forms in obstetrics and gynaecology residency selection: A retrospective cohort study on the optimal number of committee members
Source: PLoS One. 2025 Nov 19;20(11):e0336478. doi: 10.1371/journal.pone.0336478 (PMC12629435; doi:10.1371/journal.pone.0336478)
Supplement: S1 Table — (DOCX) [file pone.0336478.s006.docx]

### Supplementary Table 1 Intraclass correlations across all cohorts

|  | Cohort |  |  |
| --- | --- | --- | --- |
| Rater | 2022 | 2023 | 2024 |
| 2 | 0.739 (0.324-0.899) | 0.745 (-0.013-9.925) | 0.689 (-0.269-0.906) |
| 4 | 0.818 (0.638-0.922) | 0.827 (0.601-0.938) | 0.88 (0.76-0.947) |
| 6 | 0.889 (0.789-0.951) | 0.816 (0.596-0.933) | 0.925 (0.856-0.966) |
| 8 | 0.885 (0.786-0.949) | 0.871 (0.729-0.952) | 0.933 (0.875-0.969) |
| 10 | 0.916 (0.846-0.962) | 0.882 (0.768-0.956) | 0.934 (0.876-0.97) |
| All raters | 0.949 (0.908-0.977) | 0.947 (0.894-0.980) | 0.965 (0.938-0.984) |
